# Protein Engineering of Electron Transfer Components from Electroactive *Geobacter* Bacteria

**DOI:** 10.3390/antiox10060844

**Published:** 2021-05-25

**Authors:** Tomás M. Fernandes, Leonor Morgado, David L. Turner, Carlos A. Salgueiro

**Affiliations:** 1 UCIBIO, Chemistry Department, NOVA School of Science and Technology, Universidade NOVA de Lisboa, Campus Caparica, 2829-516 Caparica, Portugal; tmo.fernandes@campus.fct.unl.pt (T.M.F.); mlmorgado@fct.unl.pt (L.M.); 2 Instituto de Tecnologia Química e Biológica António Xavier, Universidade NOVA de Lisboa, Avenida da República (EAN), 2780-157 Oeiras, Portugal; turner@itqb.unl.pt

**Keywords:** electroactive microorganisms, Microbial Electrochemical Technologies (METs), bioenergy production, bioremediation, protein engineering, multiheme cytochromes, redox characterization, Nuclear Magnetic Resonance (NMR)

## Abstract

Electrogenic microorganisms possess unique redox biological features, being capable of transferring electrons to the cell exterior and converting highly toxic compounds into nonhazardous forms. These microorganisms have led to the development of Microbial Electrochemical Technologies (METs), which include applications in the fields of bioremediation and bioenergy production. The optimization of these technologies involves efforts from several different disciplines, ranging from microbiology to materials science. *Geobacter* bacteria have served as a model for understanding the mechanisms underlying the phenomenon of extracellular electron transfer, which is highly dependent on a multitude of multiheme cytochromes (MCs). MCs are, therefore, logical targets for rational protein engineering to improve the extracellular electron transfer rates of these bacteria. However, the presence of several heme groups complicates the detailed redox characterization of MCs. In this Review, the main characteristics of electroactive *Geobacter* bacteria, their potential to develop microbial electrochemical technologies and the main features of MCs are initially highlighted. This is followed by a detailed description of the current methodologies that assist the characterization of the functional redox networks in MCs. Finally, it is discussed how this information can be explored to design optimal *Geobacter*-mutated strains with improved capabilities in METs.

## 1. Introduction

Electroactive microorganisms have been extensively studied since their discovery more than a century ago [1] and are defined by their ability to exchange electrons between intracellular donors and extracellular acceptors [2], a phenomenon designated Extracellular Electron Transfer (EET). These microorganisms span all three domains of life (Archaea, Bacteria and Eukarya) and are capable of producing electrical current and transferring electrons to different electrode surfaces in a multitude of bioelectrochemical devices [2]. Electroactive microorganisms have developed metabolic features that allow them to thrive in extreme environments, using toxic and radioactive compounds as terminal electron acceptors [3]. These features make them interesting targets for Microbial Electrochemical Technologies (METs), ranging from bioenergy production [4,5] and bioremediation applications [3,6], to the fields of bioelectronics [7,8] and bionanotechnology [9].

In the last 15 years, METs have been intensively studied as flexible and practical platforms for the mentioned applications, which include electricity production [10], wastewater treatment and pollutant removal [11], desalination [12], production of H_2_ [13], biomethane [14] and biofuels [15], among other commodities. However, due to the low current production levels, METs are still limited to lab-scale setups, except for a few pilot-scale microbial fuel cells [16] and U(VI) bioremediation stations [17].

The development of METs depends on a better understanding of their abiotic and biotic components, which include the electrode materials [18], the reactor design and operating conditions [19], the medium or electrolyte composition [20,21] and the type of microbial catalyst [22]. From the biotic point of view, the efficacy of the electron transfer processes of the selected microbial catalysts can be optimized through several strategies that include selective adaptation [23], synthetic biology (for a review see [24,25]) and protein engineering, particularly of multiheme cytochromes (MCs), which will be the main focus of this Review.

**Figure 1:**
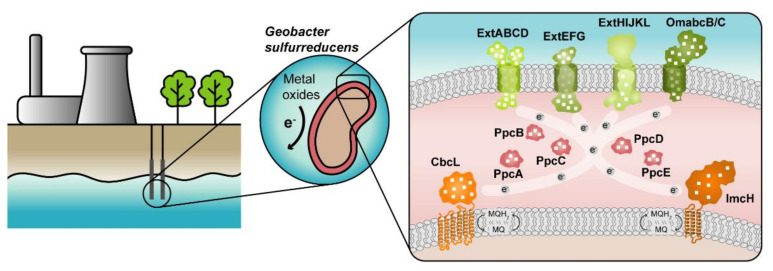
*Geobacter*-based bioremediation technologies and current model for EET in *G. sulfurreducens*. A conceptual bioremediation station installed near soils, sediments and groundwater contaminated by industrial activities is represented. In this station, organic carbon compounds are introduced to native microorganisms, such as *G. sulfurreducens*, through injection wells (see [28]). This bacterium couples the oxidation of those compounds with the reduction of different toxic or radioactive compounds, leading to their precipitation and thus facilitating their removal. On the right panel, a model for the EET pathways of *G. sulfurreducens* is presented. The different proteins that play a role in EET spread from the inner membrane (CbcL and ImcH in orange), through the periplasm (PpcA-family in pink), and to the outer membrane (porin-cytochrome complexes in green). Heme groups are represented in white.

The highest current densities recorded to date in METs come from mixed cultures that are usually dominated by Deltaproteobacteria of the genus *Geobacter* [2], such as *G. sulfurreducens*, making them the most often selected microbial catalyst for such applications. *G. sulfurreducens* was firstly classified as a strict anaerobe naturally found in a variety of soils and sediments. Later, it was discovered that it could withstand low levels of molecular oxygen, providing an explanation for its abundance in oxic subsurface environments [26]. This bacterium is able to reduce a large variety of extracellular compounds, including Fe(III), U(VI) and Mn(IV) oxides, as well as many toxic organic substances that contaminate soils and wastewaters [27] and for this reason, *Geobacter* is being explored in bioremediation technologies (Figure 1).

The genome of *G. sulfurreducens* codes for over 100 putative *c*-type cytochromes that form electron transfer networks with high flexibility and apparent redundancy [29], thus allowing the reduction of diverse terminal electron acceptors. Up to date, extensive proteomic and gene deletion studies identified different proteins as essential for the reduction of each electron acceptor [30,31,32,33,34,35,36,37,38,39,40,41,42,43,44,45,46,47,48,49,50,51].

The cytochromes from *G. sulfurreducens* are localized at the bacterium’s inner membrane, periplasm and outer membrane, allowing the transfer of electrons from intracellular carriers, such as NADH, to extracellular acceptors. The current model for EET in this bacterium (Figure 1) states that electrons from NADH are transferred to the menaquinone pool via the NADH dehydrogenase located in the inner membrane. Then, depending on the redox potential of the final electron acceptor, different proteins are involved in the quinone regeneration: the CbcL-dependent pathway operates with acceptors at or below redox potentials of 100 mV (versus the normal hydrogen electrode, NHE), whereas the ImcH-dependent pathway operates above this redox potential [41,51]. In either case, electrons are supplied to periplasmic cytochromes. The most abundant periplasmic cytochromes belong to the PeriPlasmic Cytochrome A (PpcA)-family. This family is composed of five triheme cytochromes (PpcA-E) that are responsible for traversing electrons across the periplasmic space into the outer membrane components. These outer membrane components are porin-cytochrome trans-outer membrane complexes, which are responsible for the reduction of the final extracellular terminal acceptors [35,42].

The suggested model highlights the importance of MCs in the EET mechanisms of *G. sulfurreducens*. They are logical targets for rational protein engineering focusing on the tuning of their redox properties, resulting in improved forms of these electron transfer components. Rational-mutated *Geobacter* strain, containing several optimized EET components and increased respiratory rates, can putatively contribute to more efficient METs.

**Figure 2:**
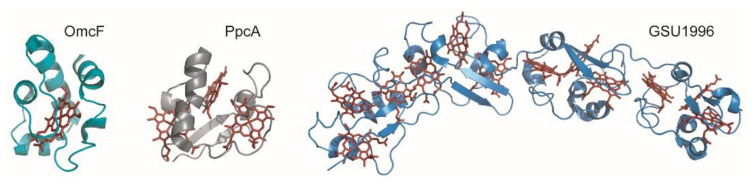
Structures of different *c*-type cytochromes from *Geobacter sulfurreducens* obtained in the oxidized state. Monoheme OmcF (PDB ID: 3CU4 [55]), triheme PpcA (lowest energy, PDB ID: 2MZ9 [56]) and dodecaheme GSU1996 (PDB ID: 3OV0 [57]). In all structures, the heme groups are represented in red.

Cytochromes *c* are proteins containing one or several *c*-type heme groups that often function as electron carriers in biological systems (Figure 2) [52,53]. They were also shown to work as electron biocapacitors, contributing to the enhancement of the electron-storage capacity of several bacteria [54]. The polypeptide chain of cytochromes is covalently bound to the heme groups through thioether linkages established with the sulfhydryl groups of two cysteine residues in a conserved binding motif sequence CXXCH, where X represents any amino acid.

MCs can receive or donate multiple electrons and protons in a cooperative way, depending on the properties of the neighboring hemes or heme surrounding protonatable centers [58,59]. Typically, the distances between the iron atoms of adjacent hemes do not exceed 15 Å, allowing fast and efficient electron exchange between the redox centers [60]. The several heme groups extend the protein’s global working redox potential ranges because of the contribution of each individual heme redox potential. The redox potential values of the hemes in MCs are affected by several factors, namely, the (i) molecular interactions that differently affect the free energy between the oxidized and reduced states; (ii) electrostatic interactions within the protein or with the solvent; (iii) heme solvent exposure; (iv) heme puckering; (v) ionization state of the heme propionate groups and (vi) nature and/or orientation of the heme axial ligands [53,61,62,63,64,65,66,67,68]. Furthermore, MCs have a lower amino acid to heme ratio compared to monoheme cytochromes, and, consequently, the heme groups are more solvent-exposed (Figure 2). The high solvent exposure and the typical His-His axial heme coordination strongly contribute to the lower redox potentials values generally observed in MCs.

Several strategies have been developed to characterize MCs, both functionally and structurally [69,70,71,72,73,74]. These strategies have been useful to reveal the functional mechanisms and key residues involved in the redox reactions of these proteins. In this Review, a description of the methodologies that allow the detailed redox characterization of the functional electron and proton networks in MCs is presented. The triheme cytochrome PpcA from *G. sulfurreducens* is used as a model to illustrate these methodologies. The knowledge obtained from such studies is currently being explored to design *Geobacter* strains with higher EET efficiency, to improve *Geobacter*-based biotechnological applications.

## 2. Thermodynamic Characterization of MCs

The characterization of the properties of the redox centers in MCs, and the concomitant elucidation of their functional mechanisms, is especially challenging due to the co-existence of several microstates in solution.

### 2.1. Redox Titrations Followed by Visible Spectroscopy

**Figure 3:**
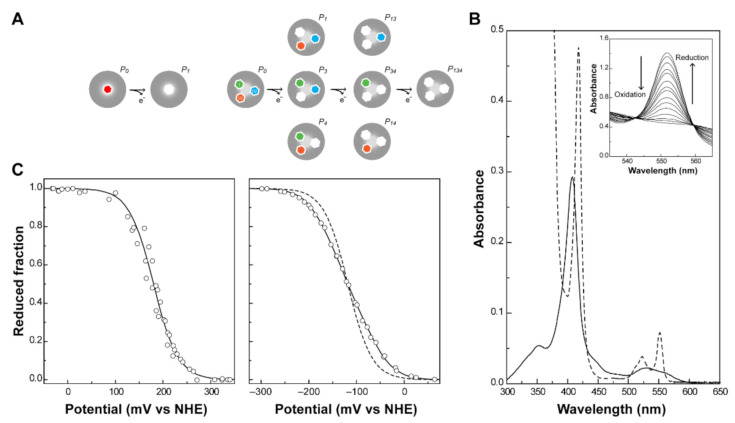
Redox features of monoheme and triheme cytochromes. (**A**) Electronic distribution scheme of a monoheme (left) and a triheme (right) cytochrome. The inner hexagons represent heme groups, which can either be reduced (colored hexagons) or oxidized (white hexagons). The microstates are grouped according to the number of oxidized hemes in each oxidation stage, connected by consecutive one-electron redox steps. *P*_0_ indicates the fully reduced state. *P_ijk_* indicates the microstates where hemes *i*, *j* and *k* are oxidized. (**B**) UV-visible spectra of a low-spin *c*-type cytochrome in the fully oxidized (solid line) and fully reduced (dashed line) states. The inset shows the α-band region of the visible spectra in the redox titration of PpcA [75]. (**C**) Redox titrations followed by visible spectroscopy of a monoheme (OmcF [55], left panel) and a triheme cytochrome (PpcA [75], right panel). Solid lines indicate the result of the fits for the Nernst equation (OmcF, see text) and for a model of three consecutive reversible redox steps between the different oxidation stages (PpcA, see text). The dashed line in the PpcA panel represents a standard *n* = 1 Nernst curve with an *E_app_* of −117 mV, illustrating the non-Nernstian redox behavior of a MC.

The number of heme groups defines the complexity of the redox network of a cytochrome. In the simplest case, a monoheme cytochrome, one reversible step of one-electron transfer, converts the fully reduced state (*P*_0_) into the fully oxidized state (*P*_1_). Therefore, only two redox stages are defined (Figure 3A, left panel) and any technique that allows monitoring of the heme oxidation can be explored to determine the redox potential of the heme group.

UV-visible spectroscopy is widely used to explore the different spectral features of heme groups. In fact, cytochromes can exist in a variety of electronic states, and the different transitions between them can be monitored by this technique. The most relevant molecular orbitals for the UV-visible spectral features of cytochromes are the π-bonding and π*-antibonding orbitals of the porphyrin. The typical spectra of a low-spin hexacoordinated *c*-type cytochrome are shown in Figure 3B. The absorption of the heme groups dominates the spectrum in both redox states. Due to π-π* transitions, the hemes originate bands at ~410 nm (Soret band, common to both redox states) and between 500 and 600 nm (β and α bands, typical of the reduced state spectrum) [53]. The β and/or α bands can be used to monitor the oxidation of the protein in UV-visible potentiometric redox titrations and hence, to study the variation of the reduced fraction of the protein with the solution potential (Figure 3C). For monoheme cytochromes, the heme reduction potential value ($E_{1}^{0}$) can be directly obtained from the fitting of a Nernst equation (Equation (1)), at the point at which the oxidized and reduced fractions are equal.

(1) $$E=E_{1}^{0}+\frac{RT}{nF}\;\ln\frac{P_{i}}{P_{0}}$$

In this equation, *E* corresponds to the solution potential; $E_{1}^{0}\;$ to the midpoint reduction potential of the cytochrome (also known as *E_app_*); *P_i_* and *P*_0_ correspond to oxidized and reduced fractions of the cytochrome. The reduced fraction can be obtained through a relationship between the number of reduced molecules and the total number of molecules in solution (Equation (2)).

(2) $$Reduced\;fraction=\frac{P_{0}}{P_{0}+P_{i}}$$

By replacing *P_i_* (from Equation (1)) in Equation (2), the reduced fraction can be described as a function of *E* and $E_{1}^{0}$ (Equation (3)), which is used to fit to the experimental data obtained from a redox titration:

(3) $$Reduced\;fraction=\frac{1}{1+e^{\left(E-E_{1}^{0}\right)\frac{F}{RT}}}$$

Experimentally, in a redox titration followed by UV-visible spectroscopy, the redox potential of the solution is measured after each addition of reducing or oxidizing agents, and, simultaneously, the redox state of the protein center(s) is monitored by the UV-visible spectrum (as shown in the inset of Figure 3B). There are several experimental details to consider in order to obtain reliable and reproducible redox titration curves. The solution potential must be sufficiently stable to assure that the recorded spectrum corresponds exactly to the measured potential, and a rapid and complete equilibration between the protein redox centers and the working electrode must be obtained. This can be achieved by the use of small organic or inorganic redox agents, known as redox mediators, that work as shuttles between the electrode and the redox centers [76]. These molecules must (i) be chemically stable and react efficiently and reversibly both with the electrode and with the protein and (ii) not form complexes or interact with the target proteins to a point where they affect their electrochemical behavior. Therefore, to properly cover the entire redox window of the target proteins, one must use a combination of several redox mediators. The appropriate concentration of mediators is a compromise between the amount necessary to ensure good equilibrium between the protein and the electrode and the optical interference caused by their UV-visible signatures. Notwithstanding, the absorbance measurements must always be corrected for the contribution of the mediators. This is achieved by simply integrating the area of the α-peak above the line connecting the respective flanking isosbestic points. Additionally, an efficient stirring of the protein solution inside the spectrophotometer cell must be maintained without perturbing the measurements. To check for hysteresis, the redox titration must be performed in the oxidative and reductive directions. In the case of proteins with expected very negative redox potentials, which are readily oxidized by molecular oxygen, the redox titrations must be acquired under strict anaerobic conditions inside a glove box.

In MCs, the number of electron transfer steps and microstates in solution increase with the number of heme groups (N). In fact, the number of microstates and oxidation stages are given by 2^N^ and N + 1, respectively. In the case of a triheme cytochrome, three one-electron transfer steps convert the fully reduced state (*P*_0_) into the fully oxidized state (*P_ijk_*), thus defining four different redox stages (Figure 3A, right panel). In these cytochromes, data obtained by UV-visible potentiometric redox titrations usually do not discriminate the individual heme redox potentials. A typical redox titration curve for a triheme cytochrome is given in Figure 3C. As depicted in Figure 3A, three macroscopic redox potentials ($E_{1}^{0}$–$E_{3}^{0}$) describe the electron transfer from the fully reduced to the fully oxidized state. However, in most cases, these potentials cannot be formally assigned to any specific heme in the protein. Few exceptions have been described in the literature, as in the case of the diheme cytochrome DHC2 from *G. sulfurreducens* [77]. In this cytochrome, the redox potentials of the two heme groups are sufficiently different (more than 60 mV) to be determined by UV-visible potentiometric redox titrations [77]. This is very unusual in MCs since most examples within this group of proteins have multiple hemes with the same type of coordination, identical optical properties and redox potential values. However, macroscopic reduction potential values can be obtained for MCs from the Nernst equations defined for each one-electron transfer step (Equations (4)–(6)).

(4) $$E=E_{1}^{0}+\frac{RT}{F}\;ln\frac{P_{i}+P_{j}+P_{k}}{P_{0}}$$

(5) $$E=E_{2}^{0}+\frac{RT}{F}\;ln\frac{P_{ij}+P_{ik}+P_{jk}}{P_{i}+P_{j}+P_{k}}$$

(6) $$E=E_{3}^{0}+\frac{RT}{F}\;ln\frac{P_{ijk}}{P_{ij}+P_{ik}+P_{jk}}$$

Using a similar approach to the one above shown for monoheme cytochromes, it is possible to describe the reduced fraction of a triheme cytochrome as a function of the solution potential (*E*) and the three macroscopic reduction potentials ($E_{1}^{0}$, $E_{2}^{0}\;$ and $E_{3}^{0}$, see Equation (7)).

(7) $$Reduced\;fraction=\frac{3+2e^{\left[\left(E-E_{1}^{0}\right)\frac{F}{RT}\right]}+e^{\left[\left(2E-E_{1}^{0}-E_{2}^{0}\right)\frac{F}{RT}\right]}}{3(1+e^{\left[\left(E-E_{1}^{0}\right)\frac{F}{RT}\right]}+e^{\left[\left(2E-E_{1}^{0}-E_{2}^{0}\right)\frac{F}{RT}\right]}+e^{\left[\left(3E-E_{1}^{0}-E_{2}^{0}-E_{3}^{0}\right)\frac{F}{RT}\right]})}$$

The three macroscopic redox potentials can then be obtained from the fitting of the experimental data from a visible redox titration (Figure 3C, right panel).

### 2.2. Redox Titrations Followed by NMR Spectroscopy

The macroscopic redox potential values contain information on the working functional ranges of MCs but are insufficient to characterize the microscopic behavior of these proteins, as well as to provide mechanistic information on their electron transfer pathways. Such data can only be obtained if the redox profile of each heme is monitored independently. In MCs displaying identical optical properties, the stepwise oxidation of the hemes from the fully reduced to the fully oxidized protein can be obtained by EXchange SpectroscopY (EXSY) Nuclear Magnetic Resonance (NMR) [73,78]. These experiments allow the observation of signals that experience chemical exchange [79]. In NMR, chemical exchange refers to any process in which a nucleus exchanges between two or more environments, yielding, for example, a change in chemical shift. In an MC, if the interconversion between microstates within the same oxidation stage (intramolecular electron exchange) is fast, and the interconversion between microstates belonging to different oxidation stages (intermolecular electron exchange) is slow on the NMR time scale, the individual heme signals can be discriminated. Several experimental conditions can be optimized to meet these requirements, namely the magnetic field strength, protein concentration, temperature and ionic strength [75,80,81].

The assignment of the heme substituents resonances, particularly the heme methyl groups, in both the reduced and oxidized states, is a crucial step in the characterization of the redox behavior of an MC since they will constitute the starting or ending point (depending on the approach) of the EXSY NMR spectra analysis. The strategies underlying this assignment are presented in the next sections.

#### 2.2.1. NMR Spectral Features of *c*-Type Cytochromes

**Figure 4:**
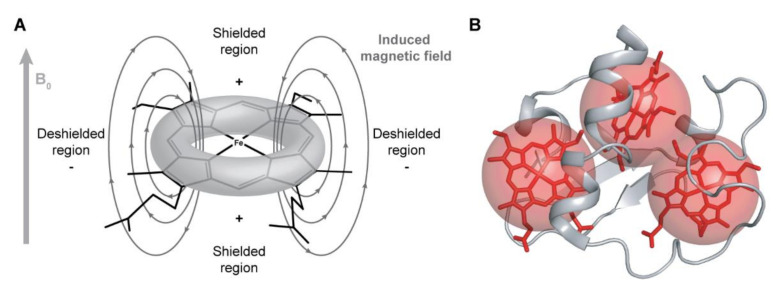
Ring-current and paramagnetic effects of the heme groups. (**A**) Model for the ring-current effects in a *c*-type heme. Based on this model, the chemical shifts of the heme substituents will be affected depending on their location in relation to the heme plane. Consequently, methines (CH), methylenes (CH_2_) and methyls (CH_3_), whose resonances are usually located between 0.9 and 2 ppm, are found in a wide range of chemical shifts, varying from −1 to 10 ppm (see below). (**B**) Paramagnetic effects in a triheme cytochrome. The paramagnetic effects (represented by red spheres) caused by the unpaired electrons do not have a linear effect on the chemical shifts of the heme substituents, which can be shifted to lower or higher frequencies on the ^1^H NMR spectrum of the cytochrome.

The NMR signals of the heme substituents are highly dependent on the ring-current and paramagnetic effects caused by the heme groups. The heme ring-current effects are caused by the circular movement of electrons around the pyrrole rings, which create local magnetic fields responsible for the typical positions observed for these signals [82]. Nuclei sensing a local magnetic field with less intensity than the applied magnetic field B_0_ (shielded regions represented in Figure 4A) are displaced to lower chemical shift values in the NMR spectrum, whereas nuclei sensing a local magnetic field with higher intensity than B_0_ (deshielded regions represented in Figure 4A) will be displaced to higher chemical shift values.

The ring-current effect is always present regardless of the oxidation state of the heme group. However, in the oxidized state, in addition to this effect, the paramagnetic contributions caused by the presence of unpaired electrons on the heme irons strongly affect the heme substituents (Figure 4B). Consequently, the same type of signals is differently affected by the paramagnetic center(s), spreading over a larger spectral window and showing different levels of broadness, making their assignment even more complex in the fully oxidized state.

**Figure 5:**
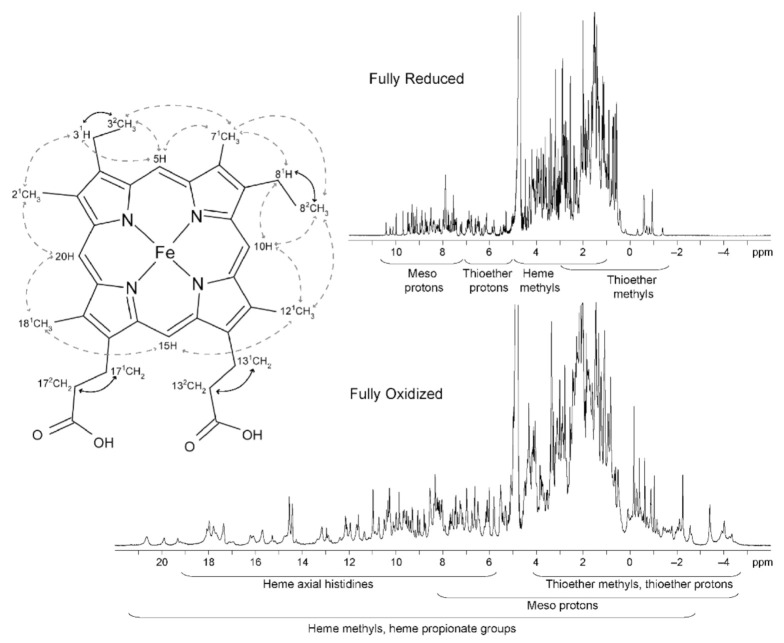
1D ^1^H-NMR spectra of the triheme cytochrome PpcA and a diagram of heme *c*. The typical regions of the hemes substituents in both redox states are indicated. In the heme *c* diagram, the heme substituents are numbered according to the IUPAC-IUB nomenclature [83]. Dashed and solid lines indicate the connectivities observed in NOESY and TOCSY spectra, respectively. This figure was partially reproduced with the permission of Elsevier from the original work [72].

The typical regions of the heme substituents of a low-spin triheme cytochrome in the reduced (diamagnetic, S = 0) and oxidized (paramagnetic, S = ½) states are illustrated in Figure 5.

In the reduced state, the signals of the heme substituents can be identified in a straightforward manner: 8–10 ppm—meso protons (5H, 10H, 15H and 20H); 6–8 ppm—thioether methines (3^1^H and 8^1^H); 2.5–5 ppm—methyl groups (2^1^CH_3_, 7^1^CH_3_, 12^1^CH_3_ and 18^1^CH_3_); and −1 to 3 ppm—thioether methyls (3^2^CH_3_ and 8^2^CH_3_). However, in the oxidized state, there are wider regions for the different heme substituents.

The heme methyl substituents are the most suitable to follow through the NMR redox titration because as the reoxidation of an MC proceeds, the resonances of these substituents become very shifted from their diamagnetic region of the spectra (Figure 5). Furthermore, the superior intensity of the heme methyl signals also simplifies their assignment.

In the next section, the different strategies that can be used to assign the heme methyl substituents of a triheme cytochrome in both redox states are introduced.

#### 2.2.2. Heme Substituents Assignment

In the reduced state, the first step of the assignment is the analysis of a 2D ^1^H-TOCSY NMR spectrum. In this experiment, scalar couplings (through bond nuclear interactions) within a spin system are observable [84], allowing the identification of the connectivities between the thioether methines (3^1^H or 8^1^H) and the thioether methyl groups (3^2^CH_3_ and 8^2^CH_3_). 2D ^1^H-NOESY NMR experiments allow the detection of spatial correlation between nuclei that are typically closer than 5 Å [84]. Meso protons have several short-range intraheme connectivities with other heme substituents (Figure 5), thus possessing distinctive patterns. Protons 20H connect with two heme methyls (2^1^CH_3_ and 18^1^CH_3_), and their identification is straightforward. Protons 5H and 10H have connectivities with a methyl group (7^1^CH_3_ or 12^1^CH_3_, respectively), a thioether methyl (3^2^CH_3_ or 8^2^CH_3_, respectively) and a thioether methine (3^1^H or 8^1^H, respectively). Consequently, their discrimination can only be achieved by observing the connectivities between the heme methyl 2^1^CH_3_ with the closest thioether groups (3^1^H or 3^2^CH_3_), which are unequivocally assigned in the 2D ^1^H-TOCSY spectrum. This allows the connection between the 20H and 5H edges of each heme. The heme methyls 7^1^CH_3_, part of the 5H edges of the hemes, show connectivities with thioether groups (8^1^H and 8^2^CH_3_), which are in 10H edges. After the identification of these three heme edges, 15H protons can be identified by observing the connectivities between cross-peaks that connect 15H and 12^1^CH_3_ or 18^1^CH_3_ protons. This strategy of assignment was applied to MCs by Turner and co-workers [85], based on the original work performed on the monoheme horse heart cytochrome *c* [86]. In the case of MCs, structural data provide information on the expected interheme NOE connectivities, which can be explored to discriminate the different heme edges and unequivocally assign the heme substituents.

**Figure 6:**
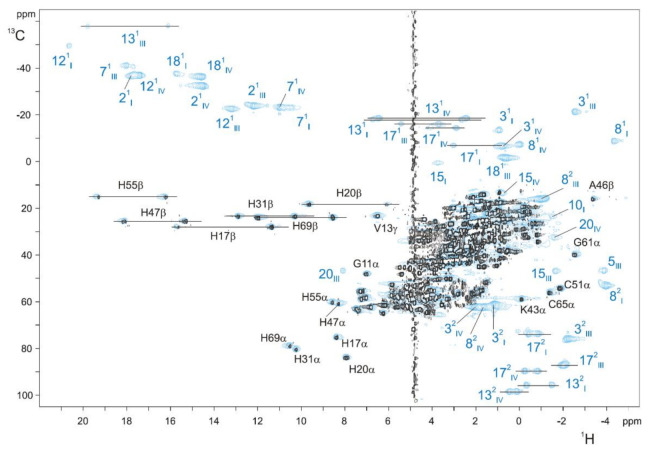
2D ^1^H,^13^C-HSQC NMR spectra of unlabeled (blue contours) and ^13^C labeled (black contours) PpcA obtained at 25 °C, with 640 and 80 scans, respectively. To not overcrowd the figure, only the resonances separated from the main signal envelope are indicated. Blue and black labels indicate the heme substituents and the polypeptide resonances, respectively. The peaks of the protons connected to the same carbon atom (CH_2_ groups) are linked by a straight line. In triheme cytochromes *c*_7_, the hemes are numbered I, III and IV [87]. This figure was reproduced with the permission of Elsevier from the original work [72].

To facilitate the assignment procedure of the heme substituents in the oxidized state, a strategy based on the combined analysis of 2D ^1^H,^13^C-HSQC NMR spectra acquired for ^13^C isotopically labeled and ^13^C natural abundance (unlabeled) samples was established, allowing for direct discrimination between the heme substituents and polypeptide chain signals [72]. Since the heme precursor (δ-aminolevulinic acid) added into the minimal media is not labeled (for more details on isotopic labeling of MCs, see [70]), the heme signals are not observable in spectra acquired with a relatively small number of accumulations in ^13^C labeled samples. Therefore, through a simple comparison of the two NMR spectra, the heme substituents signals and those of the polypeptide chain can be identified (Figure 6). After this, the specific assignment of the heme signals is obtained using the 2D ^1^H-TOCSY and 2D ^1^H-NOESY NMR spectra, as described for the reduced state [74,87,88].

#### 2.2.3. Probing the Heme Oxidation Profiles by EXSY NMR

In NMR redox titrations, samples must be in specific experimental conditions (protein and salt concentrations are the most relevant) that place the chemical exchange events of the protein at the necessary regimes. Protein samples are prepared in ^2^H_2_O to reduce the number of protein amino acid signals and placed in NMR tubes sealed with a gas-tight serum cap, from which the air is flushed out. The protein is fully reduced by the addition of catalytic amounts of the enzyme hydrogenase in the presence of gaseous hydrogen. The partially oxidized samples are obtained by the addition of controlled amounts of air in the NMR tube with a gas-tight syringe.

**Figure 7:**
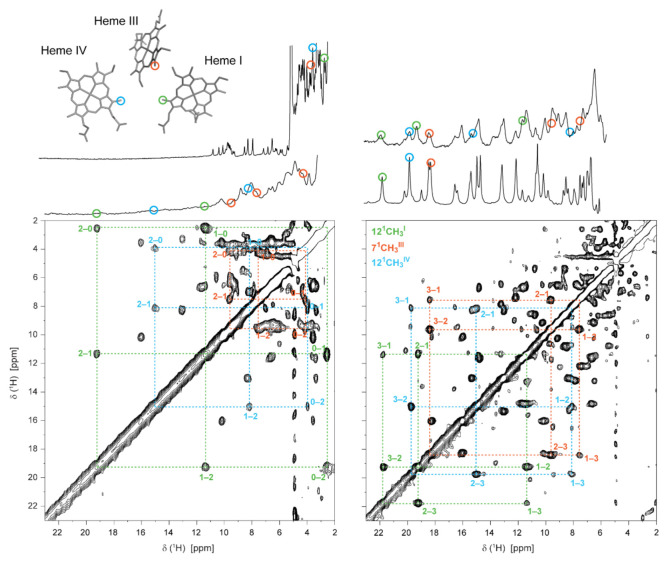
Illustration of the heme oxidation profiles of PpcA (pH 8, 15 °C). In the expansions of the 2D ^1^H-EXSY NMR spectra, the cross-peaks resulting from intermolecular electron transfer between the different oxidation stages (0–3) are indicated by dashed lines. 1D ^1^H-NMR spectra, acquired at different stages of oxidation and illustrating the redox titration of the cytochrome, are represented on top. The peaks corresponding to the heme methyls 12^1^CH_3_^I^, 7^1^CH_3_^III^ and 12^1^CH_3_^IV^ are marked by green, orange and blue circles, respectively. These heme methyls are also highlighted with the same color code in the heme core of oxidized PpcA (PDB ID: 2MZ9 [56]). This figure was partially reproduced with the permission of Elsevier from the original work [75].

The stepwise oxidation of each heme in the triheme cytochrome PpcA from *G. sulfurreducens* is illustrated in Figure 7.

The chemical shift of each heme methyl in each oxidation stage depends on the relative microscopic reduction potentials of the heme groups and thus provides information on the relative order of oxidation of the hemes [78]. The oxidation fraction of each heme *m*, in each stage of oxidation *S*, can be obtained as described in Equation (8):

(8) $$\Sigma_{m=1}^{3}\frac{\left(\delta_{obs}^{m,S}-\delta_{obs}^{m,0}\right)}{\left(\delta_{obs}^{m,3}-\delta_{obs}^{m,0}\right)}=S$$

In this equation, $\delta_{obs}^{m,S}$ corresponds to the observed chemical shift of a heme methyl *m* in the oxidation stage *S*; $\delta_{obs}^{m,0}$ corresponds to the observed chemical shift of a heme methyl *m* in the oxidation stage 0 (reduced state); $\delta_{obs}^{m,3}$ corresponds to the observed chemical shift of a heme methyl *m* in the oxidation stage 3 (oxidized state).

The oxidation fractions of PpcA at pH 8 and pH 6 are shown in Table 1. In MCs, considering the close spatial disposition of the heme groups, the paramagnetic contributions of the unpaired electrons of each heme can be intrinsic (from its own heme) or extrinsic (from neighboring hemes). The paramagnetic shifts of the heme methyls are proportional to the degree of oxidation of its heme group if the extrinsic paramagnetic shifts are negligible, as is the case of the selected heme methyls (12^1^CH_3_^I^, 7^1^CH_3_^III^ and 12^1^CH_3_^IV^), which are illustrated on the heme core of PpcA (Figure 7).

### 2.3. Redox Network

The stepwise oxidation profiles of the hemes are often modulated by the solution pH, as illustrated for PpcA in Table 1. Considering this, at each pH, a methyl group of any heme, in the oxidation stage *S*, has a single peak with a determined chemical shift ($\delta_{obs}^{i,S}$). The position of this signal is modulated by the populations of the microstates with that heme oxidized, weight-averaged according to the deprotonated and protonated populations, as represented in Equation (9).

(9) $$\delta_{obs}^{i,S}=\frac{(\delta^{i,3}-\delta^{i,0})\;\sum P_{i,S}+(\delta_{H}^{i,3}-\delta^{i,0})\;\sum P_{i,S}^{H}}{\sum P_{S}}+\delta^{i,0}$$

**Figure 8:**
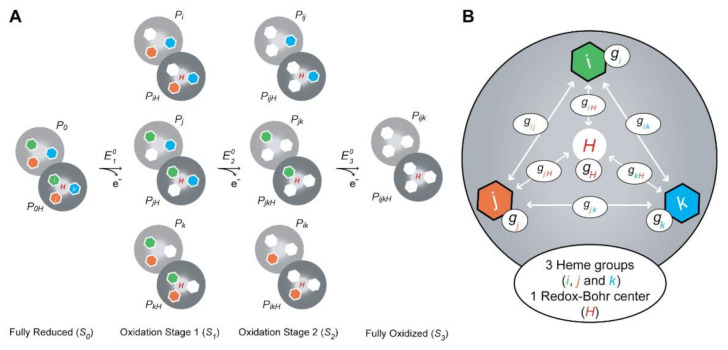
Thermodynamic model for a triheme cytochrome with one redox-Bohr center. (**A**) Electronic distribution scheme for a triheme cytochrome with a proton-linked equilibrium, showing the 16 possible microstates. The light gray and dark gray circles correspond to the deprotonated and protonated microstates, respectively. The protonated microstates are also identified with a red “H,” which mimics the redox-Bohr center. The reduced hemes *i*, *j* and *k* are colored green, orange and blue, respectively. The oxidized hemes are colored white. *P*_0_*_H_* and *P*_0_ represent the reduced protonated and deprotonated microstates, respectively. *P_ijkH_* and *P_ijk_* indicate, respectively, the protonated and deprotonated microstates, where *i*, *j* and *k* represent the heme(s) that are oxidized in that particular microstate. (**B**) Schematic representation of the interaction networks of a triheme cytochrome [inner hexagons, *i* (green), *j* (orange) and *k* (blue)] and one redox-Bohr center (red ‘H’). The terms *g_ij_* and *g_iH_* represent the interaction energies between the hemes (*ij*) and between the hemes and the redox-Bohr center (*H*), respectively. The individual heme oxidation energies are represented as *g_i_*, *g_j_* and *g_k_* for hemes *i*, *j* and *k*, respectively. This figure was partially reproduced with the permission of Portland Press from the original work [89].

In this equation, $\delta^{i,0}$ is the observed chemical shift of the methyl *i* in the reduced state, and $\delta^{i,3}$ and $\delta_{H}^{i,3}$ correspond to the chemical shifts in the fully oxidized deprotonated and protonated protein, respectively. The chemical shifts of the heme methyls in the fully reduced form are essentially unaffected by the pH and, therefore, are pH-independent (see Table 1). $\sum P_{i,S}\;$ and $\sum P_{i,S}^{H}$ are the sums over all the populations with heme *i* oxidized in stage *S*, with the protonatable center being deprotonated and protonated, respectively, whereas $\sum P_{S}\;$ is the sum over all the populations (either protonated or deprotonated) for each stage *S* (see Figure 8A).

The redox-Bohr effect tunes the heme redox potential values by changes in the protonation state of the redox-Bohr center in the vicinity of the hemes [90]. Its magnitude is determined by redox-Bohr interactions (*g_iH_*). Considering pure electrostatics, negative redox-Bohr interaction values indicate that deprotonation of the redox-Bohr center (removal of a positive charge) stabilizes the oxidized state (lower reduction potential values) and vice-versa. The addition of a redox-Bohr center into the thermodynamic model of an MC further complicates the redox network of the protein (Figure 8B). Furthermore, due to the close proximity of the heme groups in MCs, their redox potentials are mutually affected by the oxidation state of their neighbors (redox interactions, *g_ij_*). Considering pure electrostatics, the positive redox interaction values between the heme groups indicate that the oxidation of one heme hinders the oxidation of its neighbor. Therefore, with the progressive oxidation of an MC, the hemes’ redox potentials increase, and their reduced form is stabilized. The magnitude of the redox and redox-Bohr interactions correlates to the spatial distance between the interacting centers.

In conclusion, the dependence of the molar fractions of the 16 microstates (Figure 8A) across the full range of pH and solution potential can be determined by 10 thermodynamic parameters: three heme oxidation energies (reduction potential values), three interaction energies between each pair of hemes (redox interactions), three interaction energies between each heme and the redox-Bohr center (redox-Bohr interactions) and the p*K_a_* (redox-Bohr center deprotonation energy) of the redox-Bohr center (Figure 8B).

The energy of each microstate relative to the reference microstate (fully reduced with the redox-Bohr center protonated) is then given by a simple sum of the appropriate energy terms amongst the four independent centers (the three heme groups and the redox-Bohr center), the six possible two-center interactions (three redox interactions and three redox-Bohr interactions), one term that accounts for the effect of the solution potential (*SFE*) in the oxidation stage *S* (Equation (10)) and another for the proton chemical potential (*2.3RTpH*) added for the deprotonated forms (Equation (11)):

(10) $$G_{iH}=\Sigma g_{i}+\Sigma g_{ij}-SFE$$

(11) $$G_{i}=G_{iH}+g_{H}+\Sigma g_{iH}-2.3RTpH$$

In these equations, *i* and *j* represent any pair of hemes; *iH* designates a particular protonated microstate with oxidized heme(s) group(s) *i*; *g_i_* is the energy of oxidation of heme *i*; *g_ij_* is the interaction energy between each pair of hemes *i* and *j*; *g_H_* is the deprotonation energy of the fully reduced protein; *g_iH_* is the energy of interaction between the hemes and the redox-Bohr center; *S* is the oxidation stage (equivalent to the number of oxidized hemes); *F* is the Faraday constant (96,485 C mol^−1^); *E* is the redox potential of the solution; *R* is the molar gas constant (8.314 J K^−1^ mol^−1^); *T* is the absolute temperature (K).

The referred energy values can be converted to reduction potentials, which further facilitates data interpretation, using the Nernst equation (Equation (12)):

(12) ΔG=−nFΔE

In this equation, ∆*G* and ∆*E* designate the Gibbs free energy and the redox potential of a certain microstate, respectively, and *n* is the number of electrons, and *F* is the Faraday constant.

The contribution of each microstate (*P_i_*) can be determined by the Boltzmann equation (Equation (13)):

(13) $$P_{i}=e^{-G_{i}/RT}$$

The total reduced fraction can be represented as a function of the solution potential for each pH value, by accounting the relative populations weighted by the number of reduced hemes (Equation (14)):

(14) $$Reduced\;fraction=\frac{3\;\sum P_{S,0}+2\;\sum P_{S,1}+\sum P_{S,2}\;}{3\;\sum P_{S}}$$

In this equation, ∑*P_S,_*_0_, ∑*P_S,_*_1_ and ∑*P_S,_*_2_ are the sums over all the populations in stages 0, 1 and 2, respectively; $\sum P_{S}$ is the sum over all the populations in solution.

The NMR data obtained with redox titrations only defines the relative heme reduction potentials, redox and redox-Bohr interactions. Therefore, to determine the absolute potentials, the total reduced protein fractions (Equation (14)) need to be measured through redox titrations followed by UV-visible spectroscopy, as described in Section 2.1. The data obtained from both redox titrations are fitted to the presented thermodynamic model using the Marquardt method [91].

### 2.4. The Example of the Triheme Cytochrome PpcA

The redox behavior of the triheme cytochrome PpcA from *G. sulfurreducens* was thoroughly studied [75,80,92], and its detailed thermodynamic characterization is presented in this section.

**Figure 9:**
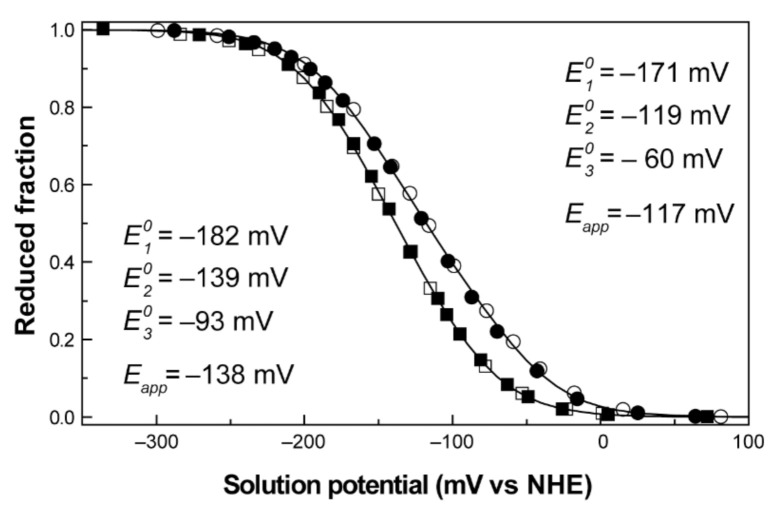
Redox titrations followed by visible spectroscopy for PpcA. The redox curves, determined at pH 7 (○) and pH 8 (□), are shown for the reductive (open symbols) and oxidative (filled symbols) titrations. The *E_app_* and $E_{1}^{0}–E_{3}^{0}$ values result from the fitting of the experimental results to Equation (7) (solid line). This figure was partially reproduced with the permission of Elsevier from the original work [93].

The redox titrations were followed by UV-visible spectroscopy at 15 °C, inside an anaerobic glove box with 0_2_ levels kept under 1 ppm, with argon circulation. The visible spectra were recorded using a spectrophotometer placed inside the anaerobic glovebox. Protein solutions were prepared with ~20 μM concentration in 80 mM sodium phosphate buffer with NaCl (250 mM final ionic strength) at pH 7 and 8. The titrations were performed at different pH values to check for pH-dependent redox behaviors (redox-Bohr effect). The solution potentials were measured using a combined Pt/Ag/AgCl electrode, calibrated with quinhydrone saturated solutions at pH 4 and pH 7. A mixture of redox mediators (gallocyanine (E^0′^ = +21 mV), methylene blue (E^0′^ = +11 mV), indigo tetrasulfonate (E^0′^ = −30 mV), indigo trisulfonate (E^0′^ = −70 mV), indigo disulfonate (E^0′^ = −110 mV), 2-hydroxy-1,4-naphthoquinone (E^0′^ = −152 mV), anthraquinone-2,6-disulfonate (E^0′^ = −184 mV), anthraquinone-2-sulfonate (E^0′^ = −225 mV), safranine O (E^0′^ = −280 mV), neutral red (E^0′^ = −325 mV), benzyl viologen (E^0′^ = −345 mV), diquat (E^0′^ = −350 mV) and methyl viologen (E^0′^ = −440 mV))** was added to the solution with a final concentration of ~2 μM to ensure equilibrium between the redox centers of the protein and the working electrode, while covering the entire redox window of the protein [75]. Furthermore, to check for hysteresis and reproducibility, each redox titration was performed in both oxidative and reductive directions, using sodium dithionite as a reducing agent and potassium ferricyanide as an oxidizing agent. The experiments were performed two times, and the reduction potentials (relative to the NHE) were found to be reproducible within ±5 mV. Each measured solution redox potential value was corrected to the NHE reference by the addition of 214 mV. The reduced fraction of PpcA was determined by integrating the area of the α-peak (552 nm) above the line connecting the flanking isosbestic points (543 and 558 nm) to subtract the optical contribution of the redox mediators. The redox titrations curves obtained for PpcA at pH 7 and pH 8 are presented in Figure 9.

No hysteresis was observed in the titration process, as the reductive and oxidative curves are superimposable, indicating that the redox process is fully reversible (Figure 9). The *E_app_* values obtained at pH 7 and 8 were −117 and −138 mV, respectively. The decrease in the reduction potential values with pH leads to a progressive stabilization of the oxidized form because of the redox-Bohr effect. These preliminary data give information on several important thermodynamic parameters, such as the working functional range of the cytochrome and the existence of the redox-Bohr effect. To obtain full mechanistic information, these data were complemented with redox titrations followed by EXSY NMR.

PpcA samples for NMR redox titrations were prepared in a concentration of 70 μM in 80 mM sodium phosphate buffer with NaCl (250 mM final ionic strength) in deuterium oxide (^2^H_2_O). Partially oxidized samples were obtained using the procedure described in Section 2.2.3.

**Figure 10:**
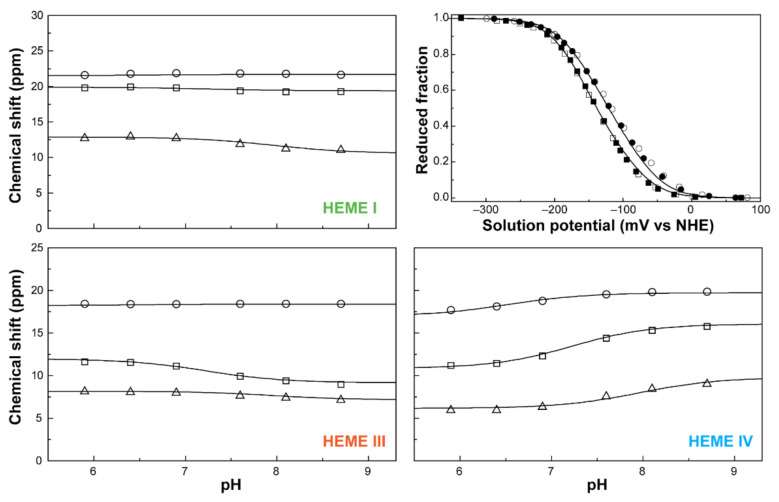
The fitting of the thermodynamic model to the experimental data for PpcA. The solid lines are the result of the simultaneous fitting of NMR and visible data. The figure shows the pH dependence of the heme methyl chemical shifts at oxidation stages 1 (Δ), 2 (□) and 3 (○), and the reduced fractions of the cytochrome, determined by visible spectroscopy at pH 7 (○) and pH 8 (□). The chemical shift dependence of the heme methyl groups in the fully reduced stage (stage 0) is not plotted since they are unaffected by the pH. In the UV-visible redox titration panel, the open and filled symbols represent the data points in the reductive and oxidative titrations, respectively. The experimental uncertainty of the NMR data is evaluated from the line width of each NMR signal at half-height, whereas the uncertainty of the UV-visible data points is usually estimated to be 3% of the total optical signal. This figure was partially reproduced with the permission of Elsevier from the original work [75].

The oxidation profiles of PpcA were studied by NMR in the pH range 5.9–8.7 [75]. At the experimental conditions used, namely 800MHz magnetic field, 15 °C and 250 mM ionic strength, it was possible to obtain well-resolved EXSY spectra and to observe and assign discrete NMR signals connecting the different oxidation stages for the heme methyl groups 12^1^CH_3_^I^, 7^1^CH_3_^III^, and 12^1^CH_3_^IV^ (Figure 6). The chemical shifts of each heme methyl at different pH values in the different oxidation stages were fitted together with the data from redox titrations, followed by visible spectroscopy to the thermodynamic model described above (Figure 10).

The thermodynamic parameters obtained from the fitting, together with the macroscopic p*K_a_* values associated with the four stages of oxidation, are presented in Table 2.

In the fully reduced and protonated form, the heme microscopic potentials are all negative. The redox interactions are positive, showing that the oxidation of one heme hampers the oxidation of the neighboring hemes.

**Figure 11:**
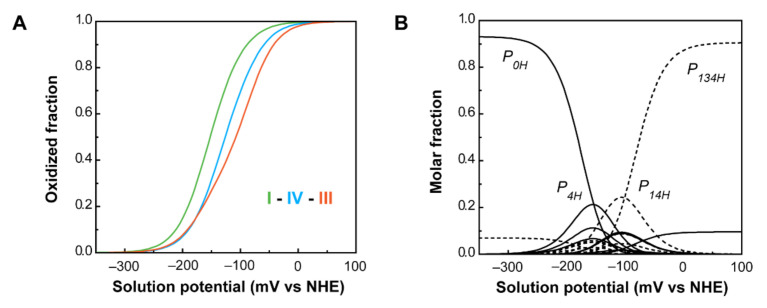
PpcA individual heme oxidation fractions and molar fractions of the 16 individual microstates at pH 7.5. (**A**) Redox dependence of the heme oxidation fractions of PpcA. The curves for hemes I, III and IV are represented in green, orange and blue, respectively. The oxidation order of the hemes is indicated. (**B**) Redox dependence of the molar fractions of the 16 microstates of PpcA. Solid and dashed lines indicate the protonated and deprotonated microstates, respectively. For clarity, only the dominant microstates are labeled. In both panels, the curves were calculated as a function of the solution reduction potential (relative to the NHE) using the parameters listed in Table 2. This figure was partially reproduced with the permission of Elsevier from the original work [75].

The interactions with the redox-Bohr center are negative, showing that the oxidation of the hemes favors the deprotonation of the protonatable center. From these parameters, the individual heme oxidation profiles, as well as the individual microstates contributions, can be determined at any pH value (Figure 11).

As a consequence of the redox and redox-Bohr interactions, at physiological pH (pH 7.5), the heme oxidation order is I-IV-III, in contrast with the potentials in the fully reduced and protonated state (I-III-IV). Moreover, the analysis of the molar fraction of the microstates in solution provides hints on the functional mechanism of the protein (Figure 11B). The forms *P*_0_*_H_* and *P*_1_*_H_* dominate PpcA oxidation stages 0 and 1, while stages 2 and 3 are dominated by the deprotonated forms *P*_14_ and *P*_134_. Therefore, a preferential pathway for electron transfer is established, and e^−^/H^+^ coupled transfer is observed between oxidation stages 1 and 2. Metabolic modelling studies in *G. sulfurreducens* showed that the cells needed additional membrane potential for ATP production when growing in the presence of insoluble electron acceptors, such as Fe(III) and Mn(IV) oxides [94]. The results obtained for PpcA showed that this protein has the necessary properties to contribute to this gradient [75].

## 3. Modulation of the Redox Properties of MC for Optimized *Geobacter* Strains

The methodology presented in the previous sections can be used for the thermodynamic characterization of key MCs of *G. sulfurreducens*. The data obtained for the wild-type proteins can then be conjugated with structural information and explored to rationally design mutated forms with optimal functional properties. In this section, an overview of MCs’ protein engineering is presented, together with a discussion on how certain mutations can be further explored in in vivo studies.

### 3.1. Enginnering of MCs—The Example of PpcA Mutants

The engineering of MCs is one of the strategies that may contribute to improve current production by electrogenic bacteria, either by increasing the bacterium’s biomass formation or by optimizing the bacterium’s electron transfer mechanisms. In the first case, mutants with enhanced e^−^/H^+^ mechanism, contributing to increased membrane potential and ATP production, can be envisaged. In the second case, the design of periplasmic proteins, with enhanced electron-transfer driving force for either upstream or downstream partners, will contribute to the creation of *Geobacter* cells with improved electron transfer capabilities.

The redox properties of the heme groups of MCs can be modulated considering different structural aspects, including intrinsic properties of the heme cofactors and those of the neighbor residues. Mutations can be designed to explore different chemical properties, including the heme’s solvent exposure and network of interactions, such as surrounding hydrogen bonds and ionizable residues. Therefore, depending on the location and nature of the targeted residue, different strategies can be applied. In this section, a summary of the different properties that have been explored to design PpcA mutants by site-directed mutagenesis is presented, together with a discussion regarding their impact on the protein’s functional behavior.

**Figure 12:**
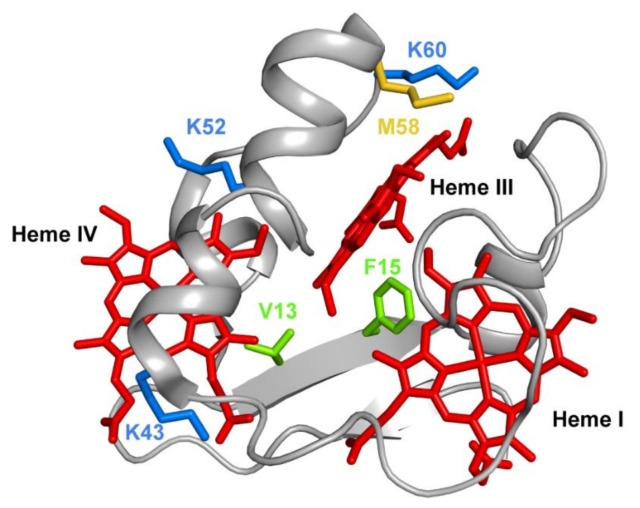
The spatial location of the residues mutated in the PpcA solution structure. The PpcA polypeptide chain (PDB code: 2MZ9 [56]) is shown as a Cα ribbon in gray, with the heme groups in red. The side chains of V13 and F15 are represented in green; K43, K52 and K60 in blue; and M58 in yellow, all in stick drawings.

Several mutations were carried out in strategic regions of the protein (Figure 12) and included replacements in (i) conserved residues found in the PpcA-family of cytochromes (V13 and F15), (ii) a residue that controls heme III’s solvent accessibility (M58) and (iii) positively charged lysine residues in the vicinity of the hemes (K43, K52 and K60).

For each mutant, the conservation of the protein’s global fold, including the spatial arrangement of the heme groups, was checked using 2D ^1^H-NOESY and 2D ^1^H,^15^N-HSQC experiments, respectively [80].

Phenylalanine 15 (F15) is an aromatic, hydrophobic residue, with a volume of 190 Å^3^, located between hemes I and III (Figure 12) and was replaced by a leucine, which is also hydrophobic but contains a smaller volume of 167 Å^3^. The results obtained showed that in the mutant, the smaller volume of the leucine’s side chain increased the solvent exposure of the nearby heme group (heme III), with the concomitant decrease of its reduction potential. The decrease of the reduction potential of heme III altered the heme oxidation order, which goes from I-IV-III in the wild-type protein to I-III-IV in the F15L mutant (Table 3), while disrupting the preferential pathway for electron transfer at physiological pH observed in the wild-type protein (Table 3).

The conserved valine residue (V13), located within van der Waals contact of hemes III and IV, was replaced by nonpolar (alanine and isoleucine) and polar (serine and threonine) residues to probe the effects of the side-chain volume and polarity [95]. Except for V13A, in all other mutants, the architecture of the heme core was significantly perturbed, indicating that V13 was naturally selected to assure a unique conformation of the protein and the order of oxidation of the hemes. Overall, the results obtained by the replacement of conserved residues at the hydrophobic heme core (V13 and F15) are not relevant for eventual mutants, since the functional properties of the wild-type cytochrome necessary to assure effectiveness in the EET respiratory pathways of *G. sulfurreducens* were no longer observed.

Methionine 58 (M58), a hydrophobic residue with a volume of 163 Å^3^, was hypothesized to control the solvent accessibility of heme III (Figure 12). To probe the influence of the local charge and heme solvent exposure in the reduction potential of this heme, different mutants were studied, including M58D, M58K and M58S. M58D contains an aspartic acid in position 58, which is negatively charged and has a smaller volume of 111 Å^3^. The presence of an extra negative charge near heme III, together with the higher solvent accessibility caused by the smaller side chain of the aspartic acid residue, lowered the reduction potential of heme III, as expected (Table 3). Overall, the mutation affected the heme III reduction potential and affected both the heme oxidation order and the e^−^/H^+^ preferential pathway for electron transfer at physiological pH (Table 3). On the other hand, in the M58K mutant, the positively charged lysine has a similar volume compared to the original residue in the wild-type cytochrome (167 Å^3^ *versus* 163 Å^3^). As expected, the effect on the heme III reduction potential value is opposite to the one observed in the M58D mutant (Table 3). In this case, the order of oxidation of the hemes is maintained, the protein is still able to couple e^−^/H^+^ transfer, and its heme’s functional redox window is larger compared to the wild-type cytochrome (−91 to −159 mV versus −108 to −152 mV).

The last mutant in position 58 (M58S) illustrates the replacement of a polar residue with a smaller side chain (89 Å^3^). In this case, a decrease in the heme reduction potential is expected, not only due to the neutral, hydrophilic intrinsic character of the serine residue, but also due to an increase in the heme solvent exposure. In fact, the heme reduction potentials are, indeed, slightly more negative when compared with the wild-type protein (Table 3), while keeping the heme oxidation order and preferential pathway for electron transfer at physiological pH.

The final example on the analysis of PpcA mutants refers to lysine residues, particularly for K43 and K52 (located near heme IV) and K60 (near heme III) (Figure 12). In each case, the positively charged lysines (with a volume of 169 Å^3^) were replaced by negatively charged (glutamic acid) and noncharged (glutamine) residues. Glutamic acid possesses a smaller negatively charged side chain (138 Å^3^), whereas glutamine is a polar, hydrophilic amino acid with a total volume of 144 Å^3^. As expected, the replacement of a positive charge by a neutral residue (glutamine) decreased the redox potential of the nearby heme, an effect that was even larger with the inclusion of a negative charge (glutamic acid) in the same position (Table 3). For both K43 mutants (K43E and K43Q), the mutations significantly affect the heme reduction potential of heme IV but with different extents. The reduction potential of the heme decreases; however, the larger variation is observed for the K43E mutant: 54 mV and 24 mV difference for K43E and K43Q, respectively. Similarly, for K52 residue, also located close to heme IV (Figure 12), the impact of the substitutions (K52E and K52Q) affected the heme IV reduction potential (Table 3). Notwithstanding, in this case, the decrease in the redox potential value in K52E (−51 mV) is not considerably higher compared to the K52Q mutant (−49 mV). The similar impact observed for these two mutants on the reduction potential of heme IV may be explained by structural interactions between the inserted glutamine and other nearby residues, including the formation of H-bonds. This is a perfect example of how rational engineering of proteins may not be completely straightforward and how particular structural rearrangements may require further optimization of nearby residues. Similar results were obtained for K60 mutants, but with a higher impact on the redox properties of heme III, the closest heme to this residue (Figure 12 and Table 3). In all lysine mutants, the working functional redox range was increased compared to the wild-type, but only the K43 and K52 mutants maintain a preferential e^−^/H^+^ transfer pathway.

**Figure 13:**
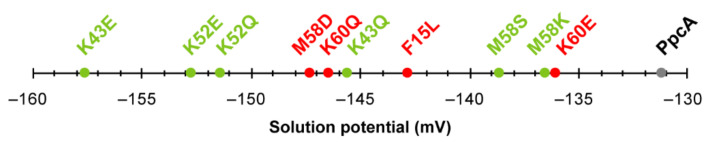
Midpoint reduction potentials (*E_app_*) of PpcA mutants. The mutants that retain the e^−^/H^+^ transfer capability are labeled in green, while mutants for which the preferential pathway is disrupted are labeled in red. Wild-type PpcA is labeled in gray.

In summary, the studies performed on the PpcA mutants showed that the replacement of residues that alter the heme solvent exposure might be explored to modify the working functional range of the proteins. Nevertheless, the most promising substitutions are indeed those involving charge alteration in the vicinity of the heme groups. Figure 13 summarizes the effect on the midpoint redox potential values (*E_app_*) of the mutants listed in Table 3.

In addition to the above-discussed effects on the properties of the individual hemes (Table 3), the analysis of Figure 13 clearly shows that all mutants have their working functional ranges shifted to more negative values and, consequently, the necessary properties to increase the electron transfer driving force towards electron acceptors in the cell exterior. The knowledge outcome from the studies carried out on PpcA cytochrome mutants [80,95,96,97,98,99] can now assist future rational optimizations of other MCs targets of *G. sulfurreducens*.

### 3.2. In Vivo Testing of the Impact of the Selected Mutations of PpcA

**Figure 14:**
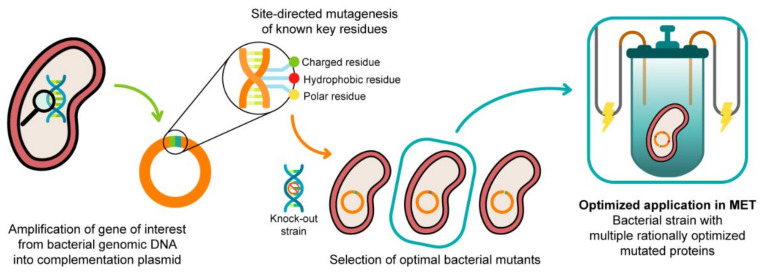
A schematic representation of the preparation of *G. sulfurreducens* strains with mutated cytochromes. After the bacterial genome is extracted, the specific target gene is amplified and further inserted into a complementation plasmid. The gene is mutated in key residues through site-directed mutagenesis, and the resultant mutants are inserted into a bacterial knock-out strain. The EET capabilities of the engineered bacteria are then tested in media with different electron acceptors. This process is applied to different key EET components of the bacterium, and the optimized mutants are selected and conjugated. The resultant strain, with higher electron transfer driving force and current production, is finally applied in METs.

The functional characterization of different PpcA mutants revealed functional features that can be tuned by single amino acid mutations, namely the ability to couple electron and proton transfer, as well as the protein redox functional ranges. These mutants might be explored in the future for increased biomass production or optimization of the electron transfer between the electron donors and acceptors. This constitutes the first step towards the rational engineering of optimized *Geobacter*-mutated strains, with optimal applicability in METs. The second step is to realistically evaluate the effect of these mutated proteins in the electron transfer capabilities of *G. sulfurreducens* when incorporated in the bacterium. In 2003, Lloyd and co-workers [100] used a *G. sulfurreducens* strain with the *ppcA* gene knocked out to show that the bacterial growth with fumarate as an electron acceptor is not affected, while the growth rate significantly decreased when Fe(III) citrate was used as an electron acceptor. The ability of the bacterium to grow with Fe (III) citrate was restored when PpcA was expressed in *trans* from a complementation plasmid inserted by electroporation [100]. A similar strategy (Figure 14) can be envisaged to probe the effects of the PpcA mutants [36,100], using either the *G. sulfurreducens* strain with the *ppcA* gene knocked out [100] or, ideally, a *G. sulfurreducens* strain with all five cytochromes from the PpcA-family knocked out [101], to allow a more straightforward interpretation of results caused by the expression of the mutated forms of PpcA.

After the production of the mutated strains, their viability must be evaluated by analyzing the growth curves in the recommended standard bacterial media for *G. sulfurreducens* (NBAF [102]), so they can later be studied in media with different electron acceptors and their mutations evaluated in terms of electron transfer efficiency and current production. However, a single mutation in a single cytochrome should not cause significant changes *per se* in the electron transfer efficiency of the bacterium.

Therefore, after the impact and viability of a certain mutation are evaluated, other mutated forms of cytochromes belonging to the same EET pathway must be inserted using a similar methodology in order to improve the directionality of the electron transfer.

### 3.3. Ongoing Biotic Strategies to Improve Extracellular Electron Transfer

The protein engineering of cytochromes can be explored as such or to complement other biotic approaches that already led to an increase in the electron transfer efficiency of electroactive microorganisms. Yi and co-workers [23] showed that appropriate selective pressures in these microorganisms could lead to the development of new strains with enhanced capacity for current production in MFC’s. In their work, a wild-type strain of *G. sulfurreducens* was inoculated in a system in which a graphite anode was poised at −400 mV (*versus* Ag/AgCl) for 5 months, from which an isolate, designated strain KN400, was later recovered. This strain possessed several phenotypic changes in the outer surface of the cell, namely a higher abundance of protein nanowires, pili and flagella, which resulted in higher current and power densities than the wild-type strain. Jensen and co-workers [103] used a synthetic-biology approach to engineer a microorganism to increase its EET properties. By inserting the porin-cytochrome outer membrane MtrCAB complex of *Shewanella oneidensis* MR-1 into *Escherichia coli*, they were able to significantly increase the capacity of the latter to reduce metal ions and solid metal oxides. More recently, Ueki and co-workers [101] trimmed the redundancy of *G. sulfurreducens* EET pathways by creating a strain with minimal requirements. This “stripped-down” strain, with well-defined EET pathways, constitutes an excellent vehicle to implement the protein-engineering strategies described in this Review, allowing the design of strains with optimal EET mechanisms.

## 4. Conclusions

METs are rapidly growing environmental technologies, bringing together several research areas, including microbiology, electrochemistry, materials science and engineering. These technologies rely on the metabolic activity of electroactive microorganisms, which oxidize and/or reduce certain compounds that could lead to the synthesis of chemicals, bioremediation of polluted matrices, treatment of contaminants of interest, as well as the recovery of energy. Despite the increasing research efforts performed in the last 20 years, these technologies are not yet available on the market, mostly because of their low conversion efficiency, limited reliability, and complex scalability. However, the knowledge obtained from model organisms such as *Geobacter* and *Shewanella* has provided details of how these microorganisms perform EET and interact with electrodes, paving the way for the usage of protein engineering as a tool to improve the electron transfer efficiency of these systems. The main protein components taking part in the EET phenomenon in *G. sulfurreducens* are MCs. The experimental and technological advances in the past decade intensified the research on MCs by facilitating the structural and functional characterization of these proteins. In this Review, the theoretical basis and state-of-the-art techniques that allow the functional redox characterization of MCs were presented, which, together with structural information, can be used to rationally design mutants and improve bioremediation and bioenergy production capabilities of the host bacteria. Indeed, several mutants obtained through site-directed mutagenesis on targeted residues located in the vicinity of the heme groups of PpcA were presented, and their impact was described in detail. Some of these mutants may be suitable to engineer optimized bacterial strains targeted to work in different functional redox ranges while keeping the same functional features. A combination of different mutated targets participating on the same EET pathways in *G. sulfurreducens* is suggested, since it may result in a bacterial strain with higher electron transfer directionality to electrodes, thus increasing the current production and the overall efficiency of METs.

## Figures and Tables

**Table 1:** Heme methyl chemical shifts of PpcA at different stages of oxidation (*S*) at pH 8 and pH 6. The heme oxidation fractions were calculated according to Equation (8).

pH 8	Chemical Shift (ppm)			*x_i_*			*∑xi*
S	12^1^CH_3_^I^	7^1^CH_3_^III^	12^1^CH_3_^IV^	12^1^CH_3_^I^	7^1^CH_3_^III^	12^1^CH_3_^IV^	
**0**	2.55	4.14	3.95	0.00	0.00	0.00	0.00
**1**	11.22	7.38	8.44	0.45	0.23	0.28	0.96
**2**	19.25	9.40	15.31	0.87	0.37	0.72	1.95
**3**	21.79	18.42	19.81	1.00	1.00	1.00	3.00
**pH 6**	**Chemical Shift (ppm)**			***x_i_***			***∑x_i_***
**S**	**12^1^CH_3_^I^**	**7^1^CH_3_^III^**	**12^1^CH_3_^IV^**	**12^1^CH_3_^I^**	**7^1^CH_3_^III^**	**12^1^CH_3_^IV^**	
**0**	2.55	4.14	3.95	0.00	0.00	0.00	0.00
**1**	12.70	8.13	5.92	0.53	0.28	0.14	0.95
**2**	19.78	11.61	11.19	0.90	0.52	0.53	1.95
**3**	21.62	18.42	17.69	1.00	1.00	1.00	3.00

**Table 2:** Thermodynamic parameters for PpcA in the fully reduced and protonated form (15 °C, 250 mM ionic strength). Diagonal values (in bold) correspond to the oxidation energies of the hemes and deprotonating energy of the redox-Bohr center. Off-diagonal values are the redox (heme-heme) and redox-Bohr (heme-proton) interaction energies. All energies are reported in meV, with standard errors given in parenthesis. The macroscopic p*K_a_* values of the redox-Bohr center in each stage of oxidation, calculated from the redox-Bohr center parameters, are the following: 8.6 (stage 0), 8.0 (stage 1), 6.9 (stage 2) and 6.5 (stage 3). The p*K_a_* of the reduced and oxidized protein is given by *g_H_F*/(2.3*RT*) and *(gH +* $\overset{3}{\underset{i=1}{\sum }}g_{iH}$)*F*/(2.3*RT*), respectively.

	Energy (meV)			
	Heme I	Heme III	Heme IV	Redox-Bohr Center
**Heme I**	**−154 (5)**	27 (2)	16 (3)	−32 (4)
**Heme III**		**−138 (5)**	41 (3)	−31 (4)
**Heme IV**			**−125 (5)**	−58 (4)
**Redox-Bohr center**				**495 (8)**

**Table 3:** Comparison of the thermodynamic properties of PpcA mutants at pH 7.5. The microscopic reduction potential values shown were calculated using the parameters listed in Table 2.

Protein	*e_app_* (mV)			Order of Heme Oxidation	Electron Transfer Pathway
	Heme I	Heme III	Heme IV		
PpcA	−152	−108	−126	I-IV-III	*P*_0_*_H_*→*P*_1_*_H_*→*P*_14_→*P*_134_
F15L	−155	−146	−125	I-III-IV	No preferential pathway
M58D	−160	−139	−140	I-(III,IV)	No preferential pathway
M58K	−159	−91	−146	I-IV-III	*P*_0_*_H_*→*P*_14_→*P*_134_
M58S	−159	−110	−139	I-IV-III	*P*_0_*_H_*→*P*_1_*_H_*→*P*_14_→*P*_134_
K43E	−165	−117	−180	IV-I-III	*P*_0_*_H_*→*P*_14_→*P*_134_
K43Q	−162	−117	−150	I-IV-III	*P*_0_*_H_*→*P*_1_*_H_*→*P*_14_→*P*_134_
K52E	−156	−116	−177	IV-I-III	*P*_0_*_H_*→*P*_14_→*P*_134_
K52Q	−157	−111	−175	IV-I-III	*P*_0_*_H_*→*P*_14_→*P*_134_
K60E	−145	−146	−119	(III,I)-IV	No preferential pathway
K60Q	−161	−143	−134	I-III-IV	No preferential pathway

## Abbreviations

**EET**	Extracellular electron transfer
**METs**	Microbial electrochemical technologies
**MC(s)**	Multiheme cytochrome(s)
